# Bootstrap confidence for molecular evolutionary estimates from tumor bulk sequencing data

**DOI:** 10.3389/fbinf.2023.1090730

**Published:** 2023-05-16

**Authors:** Jared Huzar, Madelyn Shenoy, Maxwell D. Sanderford, Sudhir Kumar, Sayaka Miura

**Affiliations:** ^1^ Institute for Genomics and Evolutionary Medicine, Temple University, Philadelphia, PA, United States; ^2^ Department of Biology, Temple University, Philadelphia, PA, United States; ^3^ Center for Excellence in Genomic Medicine Research, King Abdulaziz University, Jeddah, Saudi Arabia

**Keywords:** tumor evolution, bootstrap, bulk sequencing, metastasis, driver mutation

## Abstract

Bulk sequencing is commonly used to characterize the genetic diversity of cancer cell populations in tumors and the evolutionary relationships of cancer clones. However, bulk sequencing produces aggregate information on nucleotide variants and their sample frequencies, necessitating computational methods to predict distinct clone sequences and their frequencies within a sample. Interestingly, no methods are available to measure the statistical confidence in the variants assigned to inferred clones. We introduce a bootstrap resampling approach that combines clone prediction and statistical confidence calculation for every variant assignment. Analysis of computer-simulated datasets showed the bootstrap approach to work well in assessing the reliability of predicted clones as well downstream inferences using the predicted clones (e.g., mapping metastatic migration paths). We found that only a fraction of inferences have good bootstrap support, which means that many inferences are tentative for real data. Using the bootstrap approach, we analyzed empirical datasets from metastatic cancers and placed bootstrap confidence on the estimated number of mutations involved in cell migration events. We found that the numbers of driver mutations involved in metastatic cell migration events sourced from primary tumors are similar to those where metastatic tumors are the source of new metastases. So, mutations with driver potential seem to keep arising during metastasis. The bootstrap approach developed in this study is implemented in software available at https://github.com/SayakaMiura/CloneFinderPlus.

## 1 Introduction

Tumors are characterized by a mixture of cell populations in which many distinct clones arise due to somatic mutations (Gerlinger et al., 2012; Nik-Zainal et al., 2012; de Bruin et al., 2014; Zhao et al., 2016). These clones may increase in frequency during tumor progression, and they may spread to other locations resulting in metastasis (Gerlinger et al., 2012; Nik-Zainal et al., 2012; de Bruin et al., 2014; Zhao et al., 2016). Genetic variation in tumors is commonly profiled by bulk sequencing of tumor samples. In bulk sequencing, many cells in the sample are sequenced together to produce somatic variants and their population frequencies. This information informs the degree of genetic heterogeneity in tumors, but not the number of distinct clones present or the sequences of these clones. Knowledge of individual clone sequences is necessary to reconstruct the evolutionary relationship of tumor cells, the dynamics of mutational processes, and the history of metastatic cell migrations (Gundem et al., 2015; Wei et al., 2017; Turajlic et al., 2018; Alves et al., 2019; Kumar et al., 2020; Chen et al., 2022; Chroni et al., 2022; Miura et al., 2022).

For this reason, several methods for analyzing bulk sequencing data are available (Beerenwinkel et al., 2015; Miura et al., 2020). Some methods are designed to identify clusters of genetic variants with similar variant allele frequencies (VAFs) indicative of their co-presence in the same genotype (Roth et al., 2014; Malikic et al., 2015; Popic et al., 2015; El-Kebir et al., 2018; Xiao et al., 2020). This strategy is commonly used when bulk sequencing data from only a single sample is available. More accurate clone predictions can be achieved when multiple tumor samples are sequenced from a patient, which enables the inference of clone genotypes and their evolutionary relationships (Murugaesu et al., 2015; Hao et al., 2016; Harbst et al., 2016; Reiter et al., 2017; Martinez et al., 2018; Miura et al., 2018; Hu et al., 2019).

**FIGURE 1:**
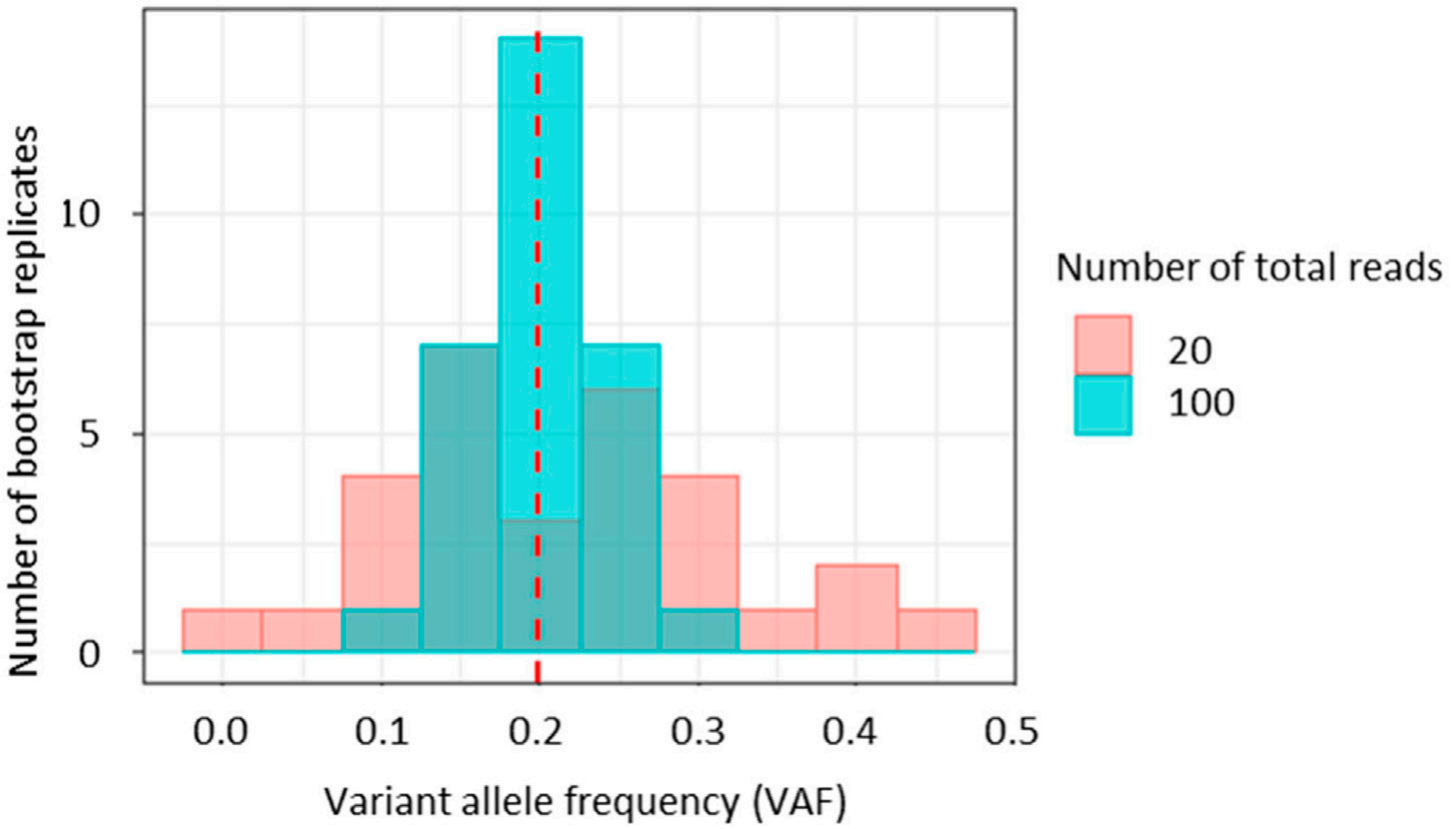
The impact of sequencing read counts on the variance of variant allele frequencies (VAFs). A variant with 20% VAF was simulated with 20 and 100 total reads. Reads were randomly sampled to generate 30 bootstrap replicates for each dataset.

Inferred clone sequences from bulk sequencing data are estimates. However, none of the current clone prediction methods provide an assessment of the uncertainty associated with these estimates. Uncertainty in clone inferences should occur because they are based on the similarities of VAFs that are calculated from observed sequencing reads with and without variants. Especially when the number of reads is small, the variance of VAF can be large (Figure 1). Thus, single nucleotide variants (SNVs) with small read counts are expected to affect the accuracy of clone prediction more strongly than those with large read counts. Unfortunately, all current methods comparing VAFs ignore this variance and simply present inferred clones and variation assignments without presenting the assignment variance. Here, we suggest using a bootstrap resampling approach to overcome this shortcoming. We have implemented this idea for use with the CloneFinder method (Miura et al., 2018) to demonstrate the usefulness of the bootstrap resampling in assessing the uncertainty of clone inference and embracing it in the downstream analysis such as the mapping of metastatic migration histories. We apply the bootstrap approach to analyze an empirical dataset, which yields insights into driver mutations and metastasis migrations.

## 2 Materials and methods

### 2.1 Bootstrap approach for tumor evolution estimates

**FIGURE 2:**
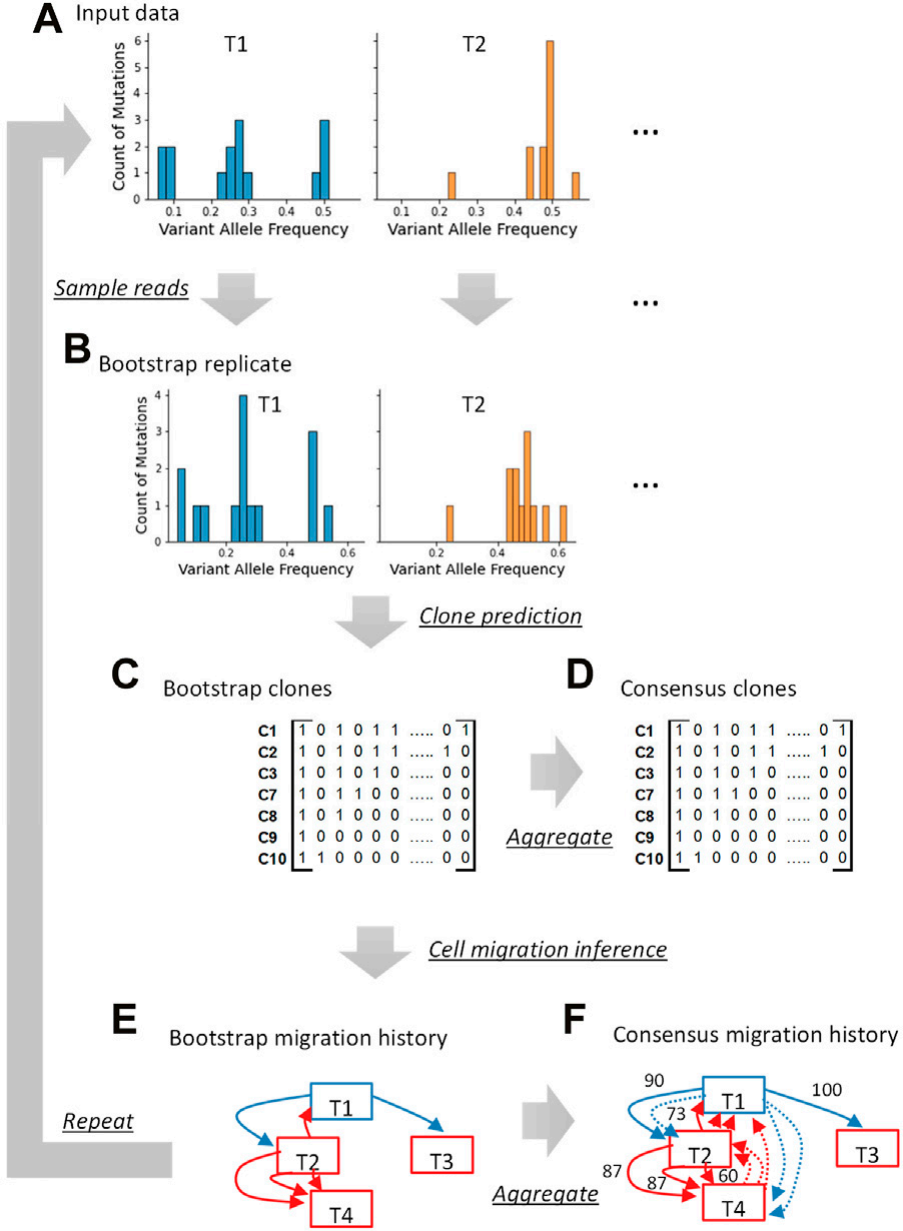
Overview of the bootstrap approach for tumor bulk sequencing data analysis. A bootstrap replicate dataset is generated by sampling reads **(A,B)**. Using a clone inference method, clones are generated from this bootstrap replicate dataset **(C)**. The presence and absence of variants are indicated with “1” and “0,” respectively. The procedure is repeated by generating many bootstrap replicates, each producing clones. All the clones from all the bootstrap replicate datasets are pooled together and consensus clones can be produced **(D)**. If a study intends to use the bootstrap approach for downstream analysis (e.g., mapping metastatic migration histories), we infer the migration history for every bootstrap replicate dataset using the phylogeny of clones inferred in that replicate **(E)**. All the migration paths inferred using bootstrap datasets are pooled and consensus migration history with bootstrap support can be generated **(F)**. The tumor sampling site is written inside the box, and the arrows indicate cell migration events. The blue boxes and blue arrows are the primary tumor sites and migration events from the primary tumor, respectively. The metastatic tumor sites and migrations from them are shown in red. A number next to an arrow is the bootstrap support (%). The dotted arrows are those with <60% bootstrap support.

Our bootstrap approach samples sequencing reads with and without variants at genomic positions (Figures 2A, B). The total number of reads sampled at a given position remains the same as in the original dataset, but reads are sampled with replacement. Since the same read can be sampled multiple times, some reads and even variants will be missing at that position in the bootstrap replicate dataset. Reads are resampled for each position, and a bootstrap replicate data is generated for each tumor sample. A pseudo-multi-tumor dataset is then generated by combining bootstrap replicates of tumor samples, and this pseudo-multi-tumor dataset is analyzed using the desired clone prediction method (e.g., CloneFinder) to infer clones (Figure 2C). Similarly, more pseudo-multi-tumor datasets are generated, and clones are inferred in many bootstrap replicates.

The bootstrap approach can be used to build consensus clone sequences. Predicted clones from all replicates are pooled together and (nearly) identical clone genotypes (for a user-supplied SNV count cutoff) are grouped (Figure 2D). Each clone group is then represented by a consensus clone and the proportion of bootstrap replicates in which a clone appears is the bootstrap support for detecting that consensus clone. To construct a consensus clone sequence, a base reconstructed in a greater proportion of bootstrap clones than the desired threshold is selected for each variant position. The base assignment is marked ambiguous when none of the bases have received the minimum desired bootstrap support.

We lastly describe an application of the bootstrap approach to infer consensus tumor evolution estimates of metastatic migration history using predicted clones. As described above, a pseudo-multi-tumor dataset is first generated by sampling reads, and a set of bootstrap clones is inferred (Figures 2A–C). These bootstrap clones are subsequently analyzed (Figure 2E). For example, we infer bootstrap cell migration history using the bootstrap clones from every bootstrap replicate dataset. Then, each migration history is expressed as a collection of individual migration paths. All the migration paths are pooled to build a consensus migration history, where paths with the highest frequency are connected with each other first, followed by others with lower frequencies until the minimum desired bootstrap support requirement is met (Figure 2F).

### 2.2 Advanced CloneFinder (CloneFinder+)

**FIGURE 3:**
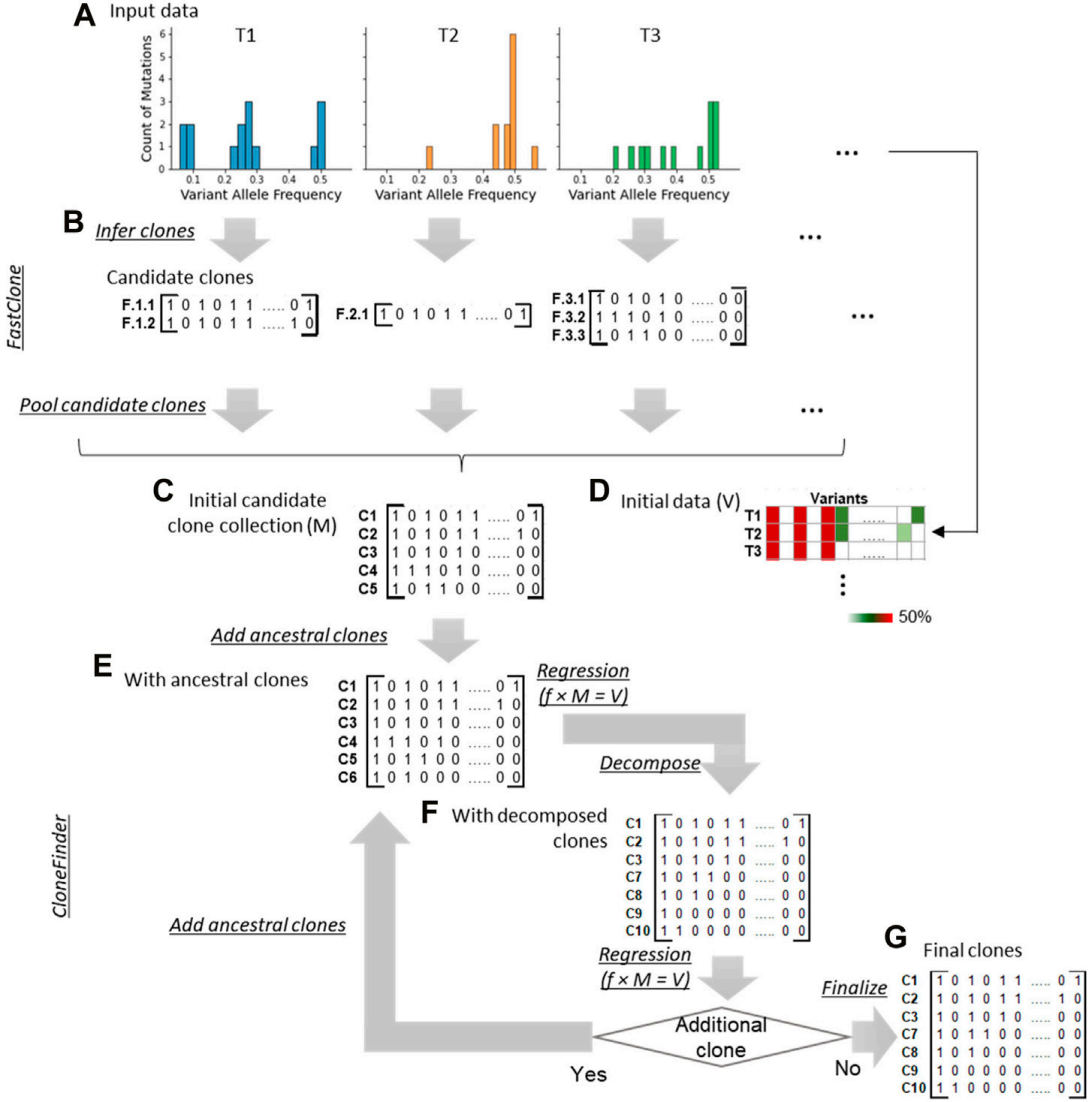
Overview of CloneFinder+. CloneFinder+ first clusters genetic variants by their VAF similarities using FastClone and then identifies candidate clones for each tumor sample **(A,B)**. Next, all candidate clones are pooled and missing clones are searched using the CloneFinder method **(C–G)**.

We implemented and tested the bootstrap approach in our CloneFinder method which is known to perform well for inferring clones using bulk-sequencing datasets from multi-tumor samples (Miura et al., 2018; Miura et al., 2020). We also took this opportunity to advance CloneFinder by adding a step to preprocess the sequencing datasets by FastClone (Xiao et al., 2020) software that clusters SNVs based on VAFs. This allows CloneFinder to start with a larger collection of tumor genotypes than the original approach. Thus, the advanced CloneFinder, CloneFinder+, begins with the analysis of VAFs using FastClone (Figure 3A). For a tumor sample, FastClone clusters SNVs based on their VAF similarities and predicts relationships of SNV clusters, i.e., ancestor-descendant, sibling, or monoclonal (Figure 3B). CloneFinder+ constructs candidate clone sequences by accumulating all predicted SNVs from the root cluster to a target cluster. These clones are the candidate clones for a given tumor sample. This analysis is done for every tumor sample individually, and candidate clones are inferred for each tumor sample. All candidate clones from all the tumor samples are then pooled and duplicate clones are removed. Also, potentially spurious candidate clone sequences are filtered, e.g., those with many ambiguous base assignments (Supplementary Note for details). Lastly, a candidate clone sequence matrix, M, is constructed (Figure 3C). This is a binary matrix, where M_*ij*_ = 0/1 represents the absence/presence of a SNV at the *j*th variant in the *i*th candidate clone.

Next, we apply CloneFinder to infer clone genotypes (Miura et al., 2018) (Figures 3C–G). A phylogeny of candidate clones is first built using the matrix M. This phylogeny is used to identify missing ancestral clones that have persisted without being replaced by their descendant clones. Ancestral clone sequences are inferred at internal nodes of the phylogeny, and all unique ancestral clones are added to the collection of candidate clones, M (Figure 3E). Then, the presence of these candidate clones in the bulk-sequencing dataset is assessed using VAFs. A matrix of VAFs, V, is constructed, where the number of rows is equal to the number of tumor samples and the number of columns is equal to the number of SNVs (Figure 3D). Next, tumor frequencies of all candidate clones are estimated through regression analysis. Here, V, M, and f, a two-dimensional matrix of estimated clone frequencies of the tumor samples, have the following relationship,

1/2f×M=V. (1)

This relationship is valid only when variants are not affected by copy number alterations (CNAs). Thus, variants that are affected by CNAs need to be excluded from the dataset; or VAFs should be adjusted before the analysis using estimated cancer cell fraction (CCF), i.e., VAF is CCF divided by two. When an estimated clone frequency within a given tumor sample is lower than the desired threshold, that clone is assumed to be absent from the tumor sample. Candidate clones predicted to be absent from all tumor samples are removed.

Next, CloneFinder + decomposes potential hybrid sequences using the strategy in CloneFinder (Miura et al., 2018) (Figure 3F). Briefly, SNVs with the same expected VAF are subclassified into two groups based on their similarity to observed VAFs. Alternate candidate clone sequences are constructed by combining SNV groups. All possible combinations of SNV groups are added to the M and are evaluated using the regression analysis (Eq. 1). All combinations estimated to be present in appreciable frequency are retained. Accordingly, M is updated, and this process of searching ancestral and hybrid clones is repeated until no new clone sequences are added. Lastly, CloneFinder+ finalizes the predicted clone sequences, where variants not assigned to any clone sequences are added to a clone based on their VAF similarities (Figure 3G).

### 2.3 Assembly of computer-simulated data

We obtained previously simulated datasets from https://github.com/raphael-group/machina. These datasets were generated by modeling the evolution of primary and metastatic tumors (El-Kebir et al., 2018). In this simulation, metastatic tumors were founded by one or more than one cancer cells that migrated from another tumor site (primary or another metastatic tumor). Clones were defined as a group of cells with the same sequences. Thus, a new clone could arise even from a single mutation. Each clone phylogeny was unique, and the number of clones was 6–26. Each dataset contained 9–99 SNVs. We excluded five datasets that contained tumor samples with only one variant because this is unrealistic in empirical data. In total, we analyzed 75 datasets.

### 2.4 Assembly of empirical data

We obtained metastatic cancer datasets from Zhao et al. (2016), who performed bulk sequencing (exome sequencing) and identified SNVs. For each SNV, these datasets contained total sequencing read counts and counts of reads with altered bases (SNVs). In total, we obtained 40 datasets with three to eight tumors.

For each SNV, we predicted if it was a driver mutation using the Cancer Genome Interpreter (CGI) web tool (http://www.cancergenomeinterpreter.org), which uses the OncodriveMUT method (Tamborero et al., 2018). We also used the CRAVAT web tool (http://www.cravat.us), which performs the CHASM prediction (Carter et al., 2009; Douville et al., 2013). Driver mutations were predicted without specifying a cancer type.

To map mutations at branches of the phylogeny, we analyzed predicted clone sequences and reconstructed ancestral clone sequences using MEGA (Kumar et al., 2012; Tamura et al., 2021).

### 2.5 Data analysis with the bootstrap approach

Each simulated and empirical dataset was analyzed using CloneFinder+, and the reliability of the inferences was assessed using the bootstrap approach. In the bootstrap analysis, we generated 30 replicates because using more (100) replicates essentially produced the same result (Supplementary Figure S1). In the CloneFinder + analysis, we clustered variants without giving the tumor purity (the step of FastClone analysis) and used variants with at least 50 reference read counts and two mutant read counts to assess the quality of candidate clones. Note that CloneFinder+ does not require the value of tumor purity and the maximum number of clones to be inferred. During the analysis, candidate clone genotypes with <1% clone frequencies for all tumor samples were discarded. We grouped identical bootstrap clones to derive consensus clone sequences while allowing at most one base assignment difference. We selected a base in >90% of bootstrap clones for each variant position.

We inferred cell migration history using the PathFinder method (Kumar et al., 2020), and the reliability was assessed using the bootstrap approach. PathFinder was performed by providing the correct primary tumor sites, sequences of CloneFinder+ clones that were predicted with >5% clone frequencies, and tumor sites that contained each clone. Note that PathFinder does not require the value of tumor purity.

### 2.6 Accuracy measurements

To evaluate the accuracy in inferring correct clones, we paired each simulated clone sequence with its most similar inferred clone sequence. We allowed an inferred clone to be paired with more than one simulated clone. We counted the number of sequence differences between inferred and simulated clones paired. We calculated the average when more than one inferred clone was paired with a given simulated clone, which was divided by the sequence length to estimate genotype error (GE) for a given simulated clone.

To evaluate the accuracy of inferred migration history, we counted the number of migration paths that were correctly inferred, those not identified, and incorrect paths following a previous study (Kumar et al., 2020).

## 3 Results

### 3.1 Bootstrap confidence for predicted clones

The bootstrap approach is widely employed in molecular evolutionary and phylogenetic analyses to estimate variances and confidence limits (Efron and Tibshirani, 1994; Nei and Kumar, 2000). Our bootstrap approach samples sequencing reads. So, VAFs will be perturbed, with greater perturbations experienced by VAFs computed from a small number of reads (Figure 1). Since the number of reads is often highly variable among SNVs in empirical data [e.g., 8–1,436 for ATP401 from Zhao et al. (2016)], the variances of their VAFs are also variable. We first show the GEs of inferred clones and their bootstrap supports.

**FIGURE 4:**
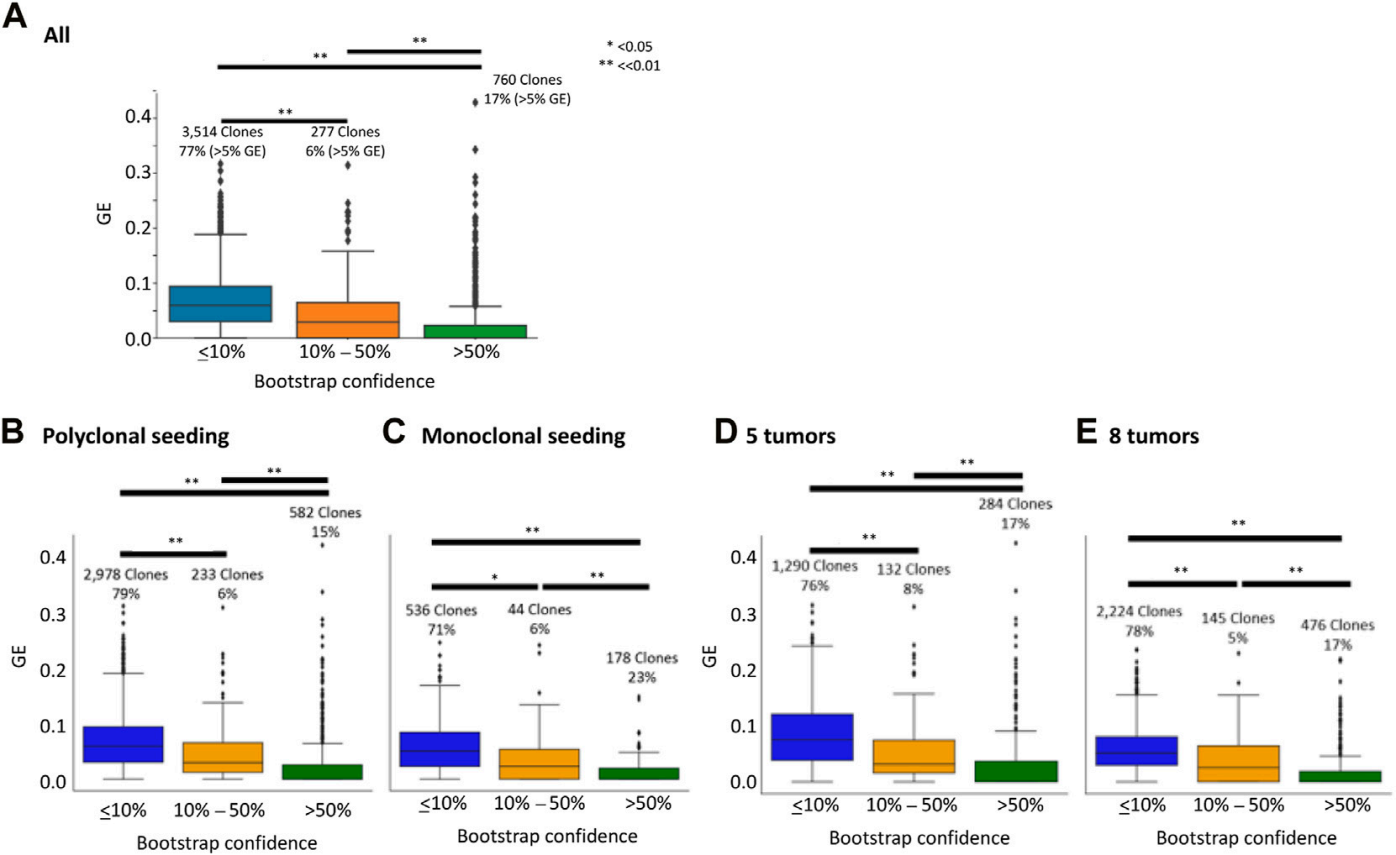
Genotype error (GE) and bootstrap supports. CloneFinder+ was used to infer clones, and the bootstrap confidence limit was calculated using the bootstrap approach. GE was computed for each clone. All datasets **(A)**, datasets with polyclonal seeding **(B)**, those with monoclonal seeding **(C)**, those with five tumors **(D)**, and those with eight tumors **(E)** were used. The number at the top of a box plot is the number of inferred clones, and the proportion of clones with >5% GE was shown at the bottom. *T*-test was performed, and the *p*-values were given, i.e., * for *p* < 0.05 and ** for *p* << 0.01. The bootstrap confidence was the proportion of replicates that produced the clone. All the simulated datasets were used.

We found that the median GE of the clones with higher bootstrap supports (>50%) was zero (Figure 4A). Therefore, clones with >50% bootstrap support can be considered to be reliably inferred. We also found that many bootstrap consensus clones (77%) received low bootstrap supports (≤10%), and these clones contained many genotype errors (GE >5%; Figure 4A). Interestingly, these clones had a wide range of GEs, even overlapping with those from higher bootstrap supports. In an extreme case, a few consensus clones with <10% bootstrap support had correct clone sequences, i.e., GE = 0. Therefore, some simulated (correct) clones were not repeatedly detected among bootstrap replicates, reducing bootstrap support.

We next tested if different scenarios of the cell migration history affected the performance of the bootstrap approach. These simulated datasets were generated by modeling the evolution of primary and metastatic tumors, where metastatic tumors were seeded by clones that migrated from either a primary or a metastatic tumor. We classified the datasets into (1) those with metastatic tumors that received a single seeding clone from another tumor site, i.e., monoclonal seeding, and (2) those with more than one seeding event, i.e., polyclonal seeding. Thus, intra-tumor heterogeneity of tumors from polyclonal seeding is higher, while inter-tumor heterogeneity among tumor sites is smaller than those with monoclonal seeding.

We found that the bootstrap approach performed well on both types of datasets, as inferred clones with greater bootstrap confidence tended to have more accurately inferred clone sequences (lower GE) (Figures 4B, C). We also found that GE tended to be slightly better for datasets with monoclonal seeding, indicating that inferred clone sequences were slightly more accurate for data with monoclonal seeding, i.e., lower intra-tumor heterogeneity with higher-inter tumor heterogeneity. This pattern was consistent with previous studies (Miura et al., 2020).

We also tested the impact of the number of tumors on the performance of the bootstrap approach. These simulated datasets were composed of five or eight tumors, i.e., four or seven metastatic tumors with a primary tumor per dataset. For both datasets with five and eight tumors, we similarly found that clones with greater bootstrap confidence tended to have more accurate clone sequences, i.e., a lower GE (Figures 4D, E). Therefore, the number of tumors in a dataset did not affect the performance of the bootstrap approach to place the reliability, while inferred clone sequences were more accurate for datasets with a larger number of tumors (lower GE). This was consistent with previous studies (Miura et al., 2020). Overall, these results suggested that the bootstrap approach is useful for assessing the reliability of inferred clone sequences.

### 3.2 Analysis of empirical data

**FIGURE 5:**
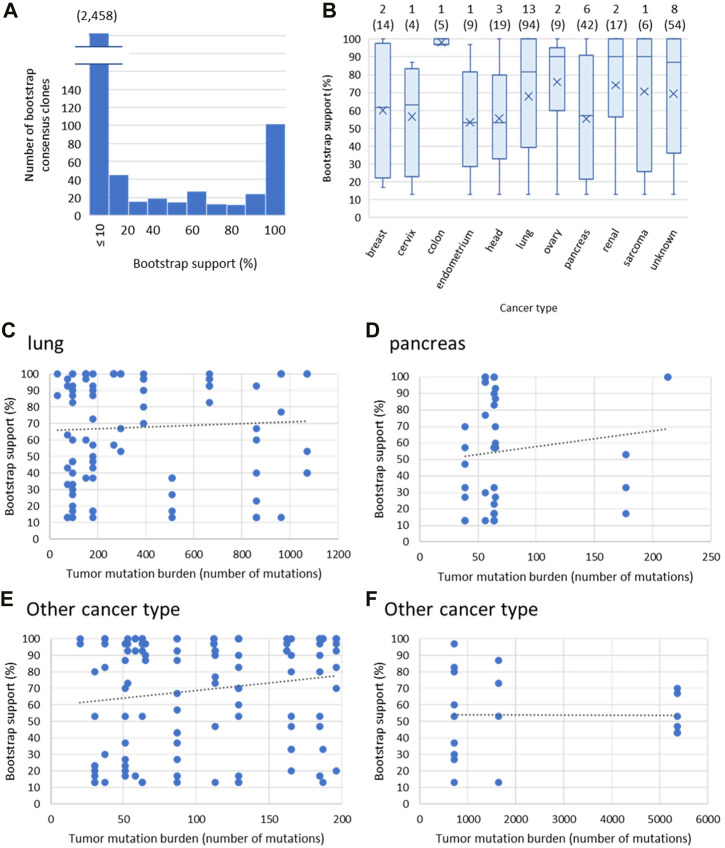
Empirical data analysis. The 40 empirical datasets from various cancer types were analyzed with the bootstrap approach. **(A)** Bootstrap support of consensus clones. All consensus clones from each dataset were pooled. The number in the parenthesis is the number of clones with ≤10% bootstrap support. **(B)** Bootstrap supports of clones for each cancer type. The cancer type is the primary tumor site. Eight datasets do not have information on the primary tumor site, so the cancer type is “unknown.” The number at the top of each box plot is the number of patients, and the total number of bootstrap consensus clones with >10% bootstrap support is shown in parenthesis. **(C–F)** The relationship between tumor mutation burden and bootstrap support of a clone for lung **(C)**, pancreas **(D)**, and other cancer with low **(E)** and high **(F)** tumor mutation burden. The tumor mutation burden is the number of total variants in a dataset. The trend line was **(C)** *y* = 0.0049*x* + 66.03 (*R* ^2^ = 0.0026), **(D)** *y* = 0.097*x* + 48.035 (*R* ^2^ = 0.018), **(E)** *y* = 0.092*x* + 59.50 (*R* ^2^ = 0.024), and **(F)** *y* = 54.17 (*R* ^2^ = 0). Clones with ≤10% bootstrap support were excluded **(B–F)**.

Next, we tested the performance of the bootstrap approach using 40 empirical datasets from various cancer types. As observed for the simulation study, most of the bootstrap consensus clones had low bootstrap supports (<10%), and few clones were identified with good (>50%) bootstrap supports (Figure 5A). We did not find a clear association between cancer types and bootstrap support values, as the distribution of bootstrap values was similar among different cancer types (Figure 5B). We also did not observe an association between tumor mutation burden (number of mutations) and bootstrap values for any cancer types (Figures 5C–F). Therefore, the performance of the bootstrap approach was not affected by either cancer type or tumor mutation burden.

### 3.3 Bootstrap confidence for inferred cell migration histories

Since the bootstrap approach performed well to place a confidence limit on inferred clones, we next tested if the bootstrap approach is also useful to assess the reliability of a downstream inference of predicted clones. As an example of a downstream analysis of predicted clones, we inferred metastatic cell migration histories using the same simulated datasets.

**FIGURE 6:**
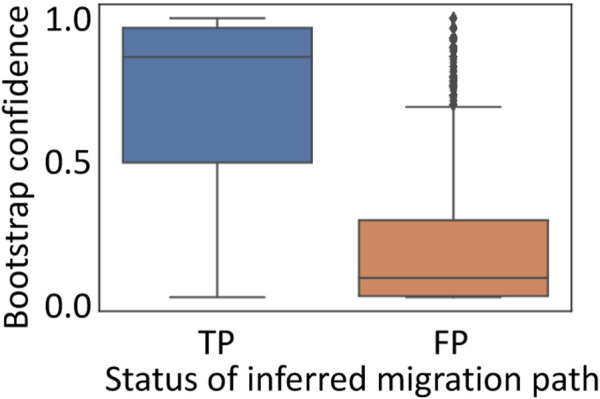
Performance of bootstrap approach to infer metastatic cell migration history. Inferred migration paths were classified into true-positive (TP; correct path) and false-positive (FP; incorrect path). All the simulated datasets were used.

We found that correct paths often had a high bootstrap support (a median bootstrap support = 86.5%), while incorrect paths tended to have low bootstrap supports (a median of 10%) (Figure 6). However, as observed in the analysis of inferred clone sequences, a few correct paths were not well supported. Actually, bootstrap support for correct paths varied considerably, indicating that these paths were not repeatedly found in many bootstrap replicate datasets. Thus, some migration paths are difficult to reconstruct, which is consistent with previous findings (Kumar et al., 2020). Overall, these results indicated that the reliability of inferred migration path from predicted clones could be assessed using the bootstrap approach.

### 3.4 Patterns of metastatic cell migrations and driver mutation occurrences

Since point estimates of clones and cell migration paths have limited accuracy, placing bootstrap confidence on inferences is essential in real empirical data analysis. As an example, we illustrate empirical data analysis for revealing the patterns of metastatic cell migrations and driver mutation occurrences. In this analysis, we excluded eight datasets without primary tumors because the information on the primary tumor site was necessary for the inference of migration history.

**FIGURE 7:**
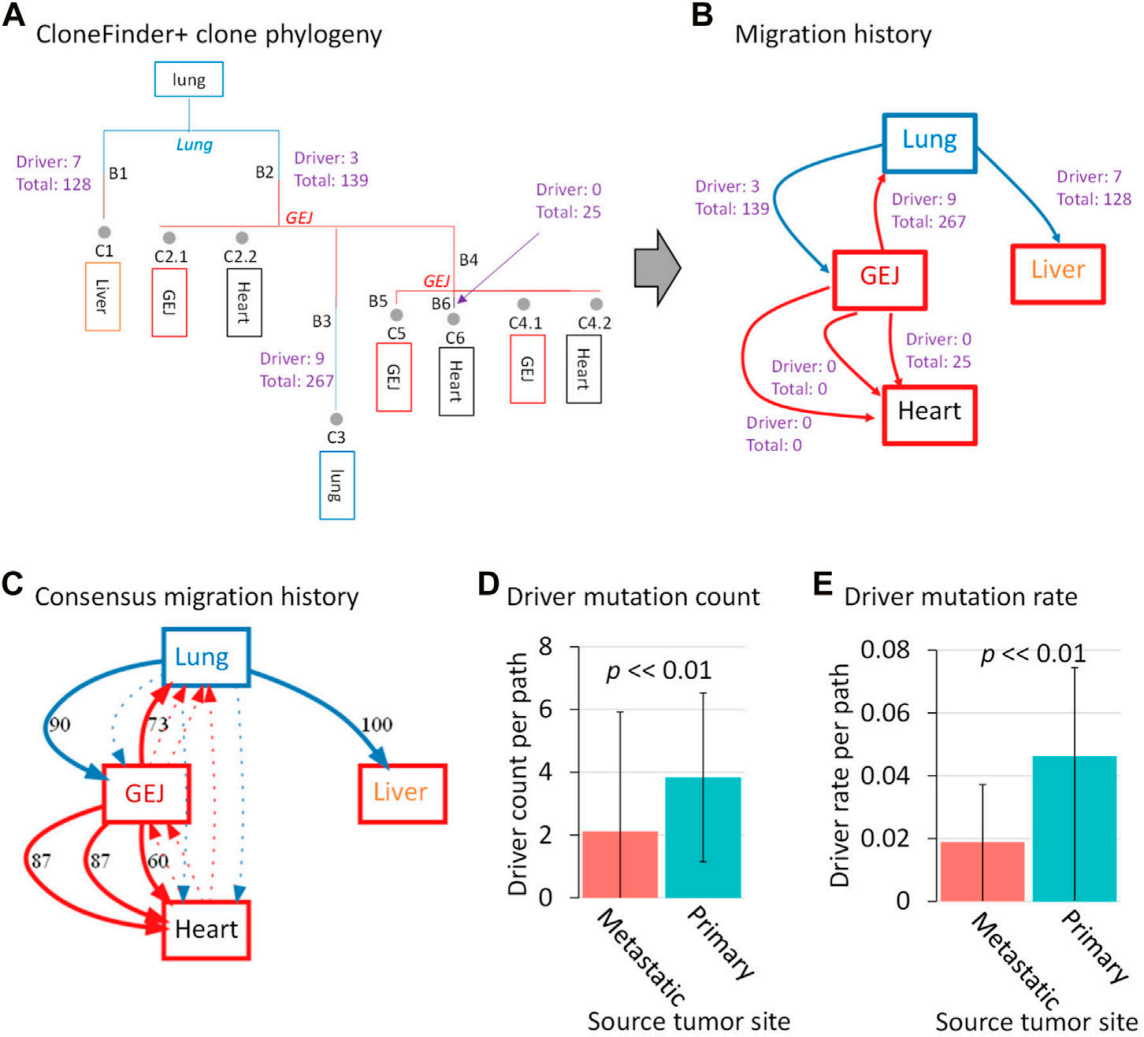
Clone phylogeny and metastatic cell migration history of a lung cancer patient. **(A)** Inferred clone phylogeny using CloneFinder+ without the bootstrap assessment. Grey circles represent tumor clones, and their predicted tumor sites (>0% clone frequency) are shown within boxes below the clone IDs. Tumor sites shown at internal nodes are predicted sites by PathFinder. Letters along branches are the branch ID, and branches are colored based on predicted tumor sites. All mutations are mapped at branches of the phylogeny through ancestral sequence reconstruction. When a cell migration event is inferred at a branch, the number of drivers and total mutations are shown. **(B)** Inferred cell migration history by PathFinder using CloneFinder+ clones without the bootstrap assessment. The numbers of drivers and total mutations are shown for each migration path. The primary and metastatic tumors are shown in blue and red boxes, respectively. **(C)** Bootstrap consensus migration history. The number along a path is bootstrap support (%). Dotted arrows indicate paths with <40% bootstrap support. **(D)** Driver mutation count and **(E)** driver mutation rates were compared between the paths originating from primary and metastatic tumors. The *p* values were computed using *t*-test. ATP401 patient was used. CGI was used for driver mutation prediction.

First, we show the result of a lung cancer patient with three metastatic tumors in the heart, liver, and GEJ (ATP401 patient). The inferred clone phylogeny using CloneFinder+ (without bootstrap reliability assessment) indicated that clone C1 originated from the root clone (the most recent common ancestor of all clones), and a metastatic tumor in the liver contained this clone (Figure 7A). The PathFinder analysis (without bootstrap reliability assessment) using these predicted clones and phylogeny produced by CloneFinder+ predicted that clone C1 migrated from the primary tumor site (lung) to the liver. While 128 mutations were mapped on the branch leading to the C1 (B1 branch in Figure 7A), the phylogeny does not tell us whether C1 acquired all, any, or a subset of new mutations in the lung. Nevertheless, seven B1 mutations were predicted to be drivers according to CGI (Tamborero et al., 2018), which may be important for metastasis. On the other hand, we also found that some migration events were not associated with any detected mutations as no mutations were mapped at corresponding branches (Figures 7A, B). For example, clone C2.1 from metastasis GEJ seeded a heart metastasis without associated mutations, as the same clone is found in both locations. Overall, we found a greater average number of driver mutations in clones that moved from the primary tumor than those from metastatic tumors for this patient (Figure 7B).

We next demonstrate how this observed pattern could be validated using the bootstrap approach. We found that all of the predicted cell migration paths in the single point PathFinder inference (Figure 7B) were supported with high bootstrap confidences (>60%), validating the PathFinder inference (Figure 7C). In the bootstrap analysis, we mapped mutations on each bootstrap migration path and calculated the average driver mutation count per path for each replicate. We found that the number of driver mutations per path was significantly greater for those from the primary tumor than those from a metastatic tumor (*p* < 0.01; *t*-test) (Figure 7D). Since migration paths with a larger number of associated mutations may simply result in a larger number of driver mutations, we normalized the count of driver mutation, i.e., we computed the driver mutation rate by dividing it by the total number of mutations for a path. We excluded paths without any associated mutations. Similarly, the driver mutation rate was significantly greater for those from the primary tumor than those from a metastatic tumor (*p* < 0.01 by *t*-test) (Figure 7E). Therefore, driver mutations occurred more frequently at migrations from the primary tumor than those from metastatic tumors for this patient.

**FIGURE 8:**
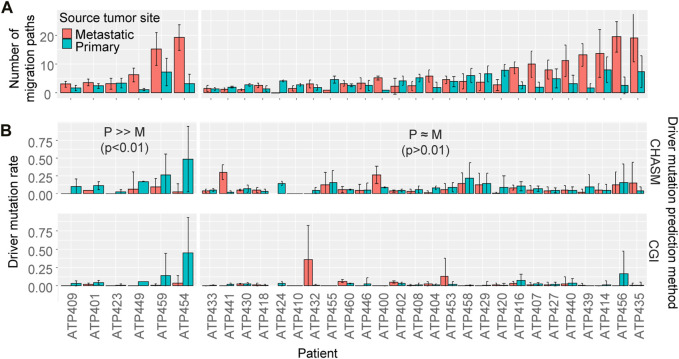
The number of cell migration paths and driver mutation rate. **(A)** The number of migration paths originating from the primary and the metastatic tumor sites. The average number among bootstrap replicates was calculated. An Error bar is the standard deviation. **(B)** The driver mutation rate of migration paths originating from the primary versus the metastatic tumor sites. The average rate among bootstrap replicates was calculated. An Error bar is the standard deviation. *t*-test was performed for each patient to test if the driver mutation rate was significantly different between those from the primary tumor and from metastatic tumors. Patients were sorted by the number of migration paths.

To test if most of the patients similarly had higher driver mutation rates for migration paths from the primary tumor than metastatic tumors, we analyzed 32 datasets of metastatic cancer patients. Similar to the ATP401 patient, many paths were from metastatic tumors (Figure 8A), indicating that migration events from metastatic tumors were not rare, consistent with previous studies (Kumar et al., 2020; Chroni et al., 2022). We found that only six patients showed significant differences in driver mutation rates between those from primary tumors and from metastatic tumors (*p* < 0.01 by *t*-test for both driver prediction methods), and all of them had higher driver mutation rates for paths from the primary tumors, which was similar to the ATP401 patient (Figure 8B). However, most of the patients (26) did not show a significant difference in driver mutation rates between migration paths from primary and metastatic tumors (*p* > 0.01 by *t*-test for at least one driver prediction method). Therefore, the numbers of driver mutations were often not significantly different between metastatic cell migration events sourced from primary tumors and those from metastatic tumors.

## 4 Discussion

In this study, we showed the potential of bootstrap resampling procedures to place confidence limits on estimates obtained from tumor sequencing data. We found that incorrect inferences tended to receive low bootstrap support. Overall, the bootstrap approach performed well to distinguish spurious inferences.

**FIGURE 9:**
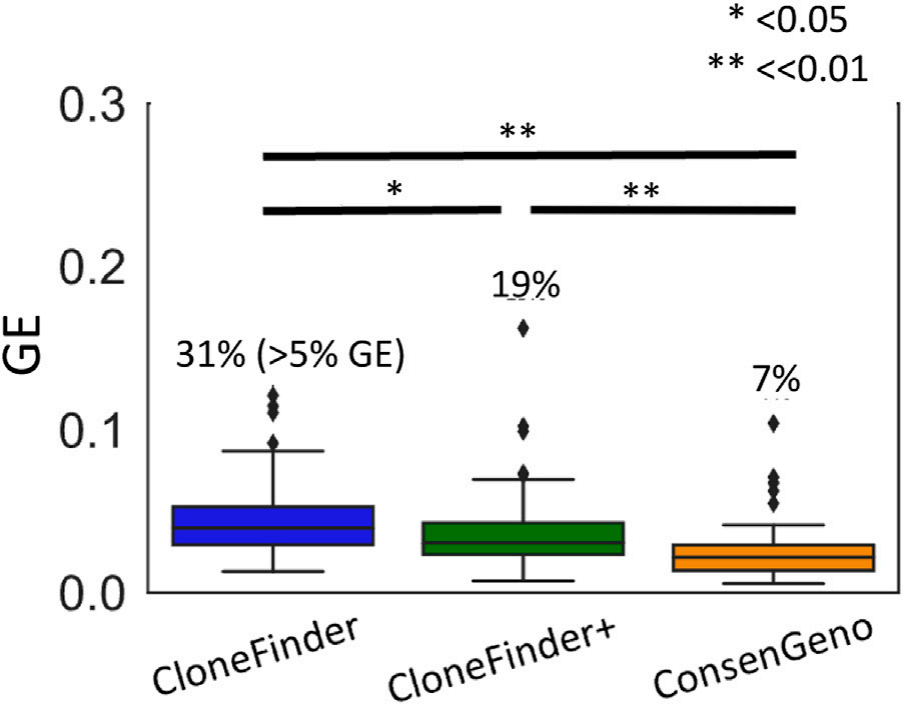
The accuracy of inferred clones by CloneFinder and single-point and bootstrap consensus inferences (ConsenGeno) by CloneFinder+. For the consensus clones, clones with <10% bootstrap support were removed. The genotype error (GE) was computed for each simulated dataset, i.e., the average over the pairs of inferred and simulated clones for a dataset. The number at the top of a box plot is the proportion of datasets with >5% GE. *t*-test was performed, and the *p*-values were given, i.e., * for *p* < 0.05 and ** for *p* << 0.01. See Supplementary Note for the parameter settings for CloneFinder. All simulated datasets were used.

Although the primary usage of the bootstrap approach is to place a confidence limit on inferred clones and downstream analysis (e.g., cell migration inferences), consensus clone sequences and consensus cell migration history can be also built by aggregating all bootstrap inferences. It is important to note that consensus inferences are not expected to be extensively more accurate than the point estimates, because the analysis of bootstrap replicates should not repeatedly produce correct inferences that are not found in the point estimate (Figure 9; Supplementary Figure S2).

In this study, we also developed CloneFinder+ by advancing CloneFinder, which now additionally analyzes the similarity of VAFs to cluster SNVs. The accuracy of CloneFinder+ was slightly better than the original version of CloneFinder (Figure 9). In conclusion, we showed that the bootstrap approach performed well to place a confidence limit on tumor evolution inference. The bootstrap approach can be coupled with any clone prediction method. Therefore, it will be useful for method developers to add a bootstrapping option.

## Data Availability

The original contributions presented in the study are included in the article/Supplementary Material, further inquiries can be directed to the corresponding author.

## References

[B1] Alves J. M. Prado-López S. Cameselle-Teijeiro J. M. Posada D. (2019). Rapid evolution and biogeographic spread in a colorectal cancer. Nat. Commun. 10, 5139. 10.1038/s41467-019-12926-8 31723138 PMC6853914

[B2] Beerenwinkel N. Schwarz R. F. Gerstung M. Markowetz F. (2015). Cancer evolution: Mathematical models and computational inference. Syst. Biol. 64, e1–e25. 10.1093/sysbio/syu081 25293804 PMC4265145

[B3] Carter H. Chen S. Isik L. Tyekucheva S. Velculescu V. E. Kinzler K. W. (2009). Cancer-specific high-throughput annotation of somatic mutations: Computational prediction of driver missense mutations. Cancer Res. 69, 6660–6667. 10.1158/0008-5472.can-09-1133 19654296 PMC2763410

[B4] Chen H.-N. Shu Y. Liao F. Liao X. Zhang H. Qin Y. (2022). Genomic evolution and diverse models of systemic metastases in colorectal cancer. Gut 71, 322–332. 10.1136/gutjnl-2020-323703 33632712 PMC8762014

[B5] Chroni A. Miura S. Hamilton L. Vu T. Gaffney S. G. Aly V. (2022). Clone phylogenetics reveals metastatic tumor migrations, maps, and models. Cancers 14, 4326. 10.3390/cancers14174326 36077861 PMC9454754

[B6] de Bruin E. C. McGranahan N. Mitter R. Salm M. Wedge D. C. Yates L. (2014). Spatial and temporal diversity in genomic instability processes defines lung cancer evolution. Science 346, 251–256. 10.1126/science.1253462 25301630 PMC4636050

[B7] Douville C. Carter H. Kim R. Niknafs N. Diekhans M. Stenson P. D. (2013). Cravat: Cancer-related analysis of variants toolkit. Bioinformatics 29, 647–648. 10.1093/bioinformatics/btt017 23325621 PMC3582272

[B8] Efron B. Tibshirani R. J. (1994). An introduction to the bootstrap. Florida: CRC Press.

[B9] El-Kebir M. Satas G. Raphael B. J. (2018). Inferring parsimonious migration histories for metastatic cancers. Nat. Genet. 50, 718–726. 10.1038/s41588-018-0106-z 29700472 PMC6103651

[B10] Gerlinger M. Rowan A. J. Horswell S. Math M. Larkin J. Endesfelder D. (2012). Intratumor heterogeneity and branched evolution revealed by multiregion sequencing. N. Engl. J. Med. 366, 883–892. 10.1056/nejmoa1113205 22397650 PMC4878653

[B11] Gundem G. Van Loo P. Kremeyer B. Alexandrov L. B. Tubio J. M. C. Papaemmanuil E. (2015). The evolutionary history of lethal metastatic prostate cancer. Nature 520, 353–357. 10.1038/nature14347 25830880 PMC4413032

[B12] Hao J.-J. Lin D.-C. Dinh H. Q. Mayakonda A. Jiang Y.-Y. Chang C. (2016). Spatial intratumoral heterogeneity and temporal clonal evolution in esophageal squamous cell carcinoma. Nat. Genet. 48, 1500–1507. 10.1038/ng.3683 27749841 PMC5127772

[B13] Harbst K. Lauss M. Cirenajwis H. Isaksson K. Rosengren F. Törngren T. (2016). Multiregion whole-exome sequencing uncovers the genetic evolution and mutational heterogeneity of early-stage metastatic melanoma. Cancer Res. 76, 4765–4774. 10.1158/0008-5472.can-15-3476 27216186

[B14] Hu X. Fujimoto J. Ying L. Fukuoka J. Ashizawa K. Sun W. (2019). Multi-region exome sequencing reveals genomic evolution from preneoplasia to lung adenocarcinoma. Nat. Commun. 10, 2978. 10.1038/s41467-019-10877-8 31278276 PMC6611767

[B15] Kumar S. Chroni A. Tamura K. Sanderford M. Oladeinde O. Aly V. (2020). PathFinder: Bayesian inference of clone migration histories in cancer. Bioinformatics 36, i675–i683. 10.1093/bioinformatics/btaa795 33381835 PMC7773489

[B16] Kumar S. Stecher G. Peterson D. Tamura K. (2012). MEGA-CC: Computing core of molecular evolutionary genetics analysis program for automated and iterative data analysis. Bioinformatics 28, 2685–2686. 10.1093/bioinformatics/bts507 22923298 PMC3467750

[B17] Malikic S. McPherson A. W. Donmez N. Sahinalp C. S. (2015). Clonality inference in multiple tumor samples using phylogeny. Bioinformatics 31, 1349–1356. 10.1093/bioinformatics/btv003 25568283

[B18] Martinez P. Mallo D. Paulson T. G. Li X. Sanchez C. A. Reid B. J. (2018). Evolution of Barrett’s esophagus through space and time at single-crypt and whole-biopsy levels. Nat. Commun. 9, 794. 10.1038/s41467-017-02621-x 29476056 PMC5824808

[B19] Miura S. Gomez K. Murillo O. Huuki L. A. Vu T. Buturla T. (2018). Predicting clone genotypes from tumor bulk sequencing of multiple samples. Bioinformatics 34, 4017–4026. 10.1093/bioinformatics/bty469 29931046 PMC6247940

[B20] Miura S. Vu T. Choi J. Townsend J. P. Karim S. Kumar S. (2022). A phylogenetic approach to study the evolution of somatic mutational processes in cancer. Commun. Biol. 5, 617. 10.1038/s42003-022-03560-0 35732905 PMC9217972

[B21] Miura S. Vu T. Deng J. Buturla T. Oladeinde O. Choi J. (2020). Power and pitfalls of computational methods for inferring clone phylogenies and mutation orders from bulk sequencing data. Sci. Rep. 10, 3498. 10.1038/s41598-020-59006-2 32103044 PMC7044161

[B22] Murugaesu N. Wilson G. A. Birkbak N. J. Watkins T. McGranahan N. Kumar S. (2015). Tracking the genomic evolution of esophageal adenocarcinoma through neoadjuvant chemotherapy. Cancer Discov. 5, 821–831. 10.1158/2159-8290.cd-15-0412 26003801 PMC4529488

[B23] Nei M. Kumar S. (2000). Molecular Evolution and phylogenetics. New York: Oxford University Press.

[B24] Nik-Zainal S. Van Loo P. Wedge D. C. Alexandrov L. B. Greenman C. D. Lau K. W. (2012). The life history of 21 breast cancers. Cell 149, 994–1007. 10.1016/j.cell.2012.04.023 22608083 PMC3428864

[B25] Popic V. Salari R. Hajirasouliha I. Kashef-Haghighi D. West R. B. Batzoglou S. (2015). Fast and scalable inference of multi-sample cancer lineages. Genome Biol. 16, 91. 10.1186/s13059-015-0647-8 25944252 PMC4501097

[B26] Reiter J. G. Makohon-Moore A. P. Gerold J. M. Bozic I. Chatterjee K. Iacobuzio-Donahue C. A. (2017). Reconstructing metastatic seeding patterns of human cancers. Nat. Commun. 8, 14114. 10.1038/ncomms14114 28139641 PMC5290319

[B27] Roth A. Khattra J. Yap D. Wan A. Laks E. Biele J. (2014). PyClone: Statistical inference of clonal population structure in cancer. Nat. Methods 11, 396–398. 10.1038/nmeth.2883 24633410 PMC4864026

[B28] Tamborero D. Rubio-Perez C. Deu-Pons J. Schroeder M. P. Vivancos A. Rovira A. (2018). Cancer Genome Interpreter annotates the biological and clinical relevance of tumor alterations. Genome Med. 10, 25. 10.1186/s13073-018-0531-8 29592813 PMC5875005

[B29] Tamura K. Stecher G. Kumar S. (2021). MEGA11: Molecular evolutionary genetics analysis version 11. Mol. Biol. Evol. 38, 3022–3027. 10.1093/molbev/msab120 33892491 PMC8233496

[B30] Turajlic S. Xu H. Litchfield K. Rowan A. Chambers T. Lopez J. I. (2018). Tracking cancer evolution reveals constrained routes to metastases: TRACERx renal. Cell 173, 581–594.e12. 10.1016/j.cell.2018.03.057 29656895 PMC5938365

[B31] Wei Q. Ye Z. Zhong X. Li L. Wang C. Myers R. E. (2017). Multiregion whole-exome sequencing of matched primary and metastatic tumors revealed genomic heterogeneity and suggested polyclonal seeding in colorectal cancer metastasis. Ann. Oncol. 28, 2135–2141. 10.1093/annonc/mdx278 28911083 PMC5834069

[B32] Xiao Y. Wang X. Zhang H. Ulintz P. J. Li H. Guan Y. (2020). FastClone is a probabilistic tool for deconvoluting tumor heterogeneity in bulk-sequencing samples. Nat. Commun. 11, 4469. 10.1038/s41467-020-18169-2 32901013 PMC7478963

[B33] Zhao Z.-M. Zhao B. Bai Y. Iamarino A. Gaffney S. G. Schlessinger J. (2016). Early and multiple origins of metastatic lineages within primary tumors. Proc. Natl. Acad. Sci. U. S. A. 113, 2140–2145. 10.1073/pnas.1525677113 26858460 PMC4776530

