# Supplementary material for: Bootstrap confidence for molecular evolutionary estimates from tumor bulk sequencing data
Source: Front Bioinform. 2023 May 16;3:1090730. doi: 10.3389/fbinf.2023.1090730 (PMC10228696; doi:10.3389/fbinf.2023.1090730)
Supplement: Supplementary file 1 [file DataSheet1.PDF]

Supplementary Material

1 Supplementary Note

1.1 Generating candidate clone genotypes from predicted variant clusters using FastClone

FastClone estimates the probability of SNV cluster assignment for each variant. Each SNV cluster is constructed by collecting variants with >99% estimated probabilities, and a variant with <1% probability is assumed to be absent from a given cluster. An ambiguous base is assigned for a variant with a probability between 1% and 99%.

FastClone also predicts the evolutionary relationship of SNV clusters. For SNV clusters in an ancestor-descendant relationship, the descendant cluster was excluded because sibling clusters were often incorrectly predicted to be ancestor-descendant clusters in our simulation study. For predicted sibling clusters, only tip (descendant) clones are generated because the presence of the ancestral clone is not assessed in the FastClone analysis, i.e., a by-product. Clone genotypes that have a larger number of ambiguous bases than the number of mutations, i.e., limited resolution, are not included in the collection of candidate clones. When all clones are excluded for a tumor sample or when FastClone has predicted only a single cluster, the genotype of the tumor sample is used as the candidate clone for a sample, i.e., the predicted monoclonal sample. To generate a tumor genotype, all variants with VAFs greater than zero are assumed to be present.

1.2 CloneFinder analysis

CloneFinder was performed using variants with at least 50 reference read counts and two mutant read counts. We discarded clones when estimated clone frequencies were <1%.

2 Supplementary Figures

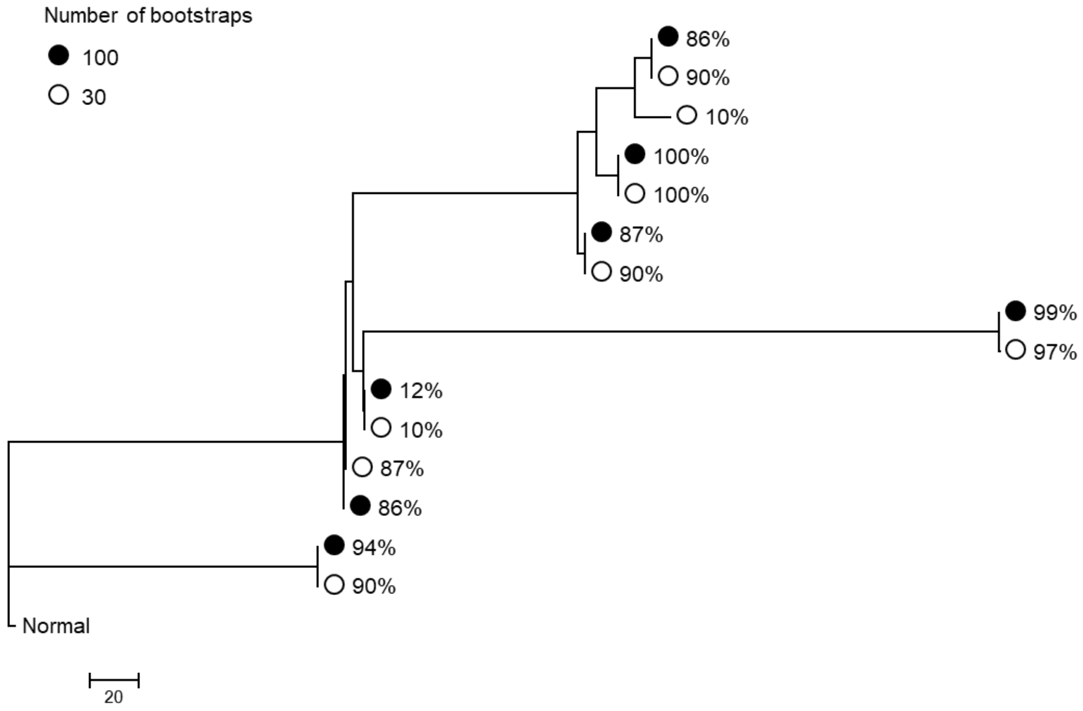

Supplementary Figure 1. Comparison of consensus clones between 30 and 100 bootstraps. Neighbor-joining phylogeny of clones from 30 and 100 bootstraps was inferred using MEGA. To compute the distance, the number of nucleotide differences was calculated. The branch lengths represent the numbers of nucleotides, and the circles are the clones, where filled and unfilled circles are those from 100 and 30 bootstraps, respectively. Similar clones were generally identified between 100 and 30 bootstraps. Clones with <10% bootstrap support were excluded. ATP401 dataset was used.

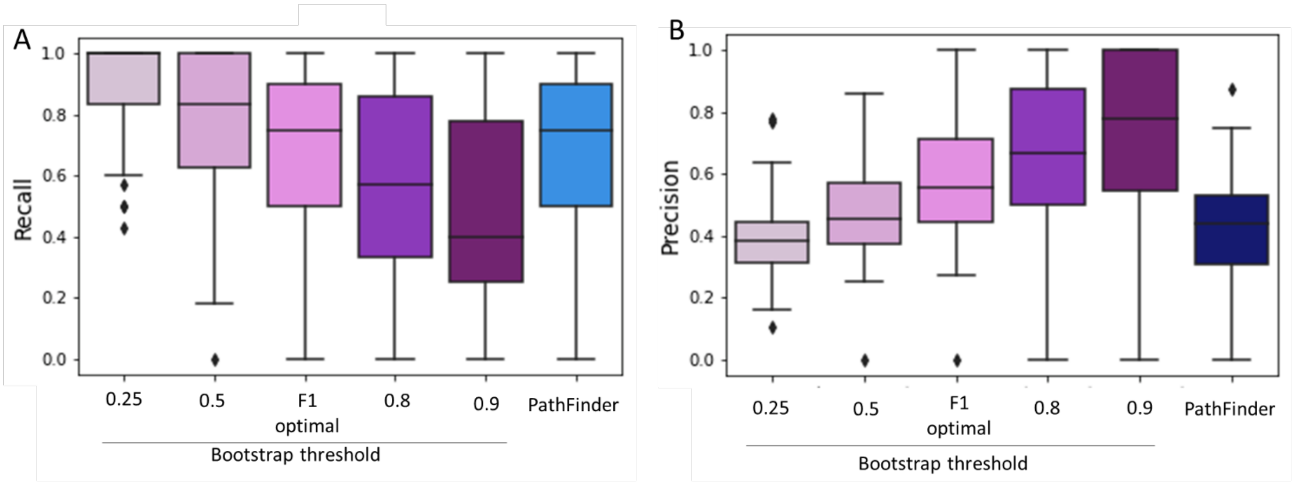

Supplementary Figure 2. Performance of bootstrap approach to infer metastatic cell migration history. Consensus metastatic cell migration history was inferred by coupling the bootstrap approach with CloneFinder+ and PathFinder. (**A**) The percent of inferred paths that are true (recall). (**B**) The percent of true paths inferred (precision). The recall and precision rates were calculated with various bootstrap thresholds. As expected, when we used a less stringent threshold (lower bootstrap support cutoff value) to filter bootstrap consensus paths, the number of correct paths inferred increased, resulting in a better recall rate. On the other hand, the number of erroneous paths inferred also increased, i.e., worse precision. Overall, the use of a 64% bootstrap support threshold maximized the F1 score, which is the average of recall and precision of inferred path accuracy. With the F1 optimal bootstrap threshold (64%) to filter migration paths, the precision rate was better than the single-point inference by PathFinder, while the recall rates were similar. This result suggested that the bootstrap approach can distinguish spuriously identified paths but cannot identify additional correct paths with high bootstrap supports.
